# Functional capacity of reconstituted blood in 1:1:1 versus 3:1:1 ratios: A thrombelastometry study

**DOI:** 10.1186/s13049-014-0080-0

**Published:** 2015-01-09

**Authors:** Arne Driessen, Nadine Schäfer, Ursula Bauerfeind, Sigune Kaske, Carolin Fromm-Dornieden, Ewa K Stuermer, Marc Maegele

**Affiliations:** Institute for Research in Operative Medicine, Faculty of Health, Department of Medicine, Witten/Herdecke University, Ostmerheimer Str. 200, D-51109 Cologne, Germany; Department of Traumatology, Orthopaedic Surgery and Sports Traumatology, Cologne-Merheim Medical Centre (CMMC), Witten/Herdecke University, Campus Cologne-Merheim, Ostmerheimer Str. 200, D-51109 Cologne, Germany; Institute of Transfusion Medicine Cologne-Merheim Medical Centre (CMMC), Witten/Herdecke University, Campus Cologne-Merheim, Cologne (Germany), Ostmerheimer Str. 200, D-51109 Cologne, Germany

**Keywords:** Transfusion practices, Acute bleeding injury, Coagulopathy, Thrombelastometry in trauma, Ratio of components

## Abstract

**Introduction:**

Different transfusion ratio concepts of packed red blood cells (pRBCs), fresh frozen plasma (FFP) and platelets (PLTs) have been implemented in trauma care, but the optimal ratios are still discussed. In this study the hemostatic potential of two predefined ratios was assessed by using an *in vitro* thrombelastometric approach. Furthermore, age effects of reconstituted blood were analyzed.

**Methods:**

Whole blood (WB) of voluntary donors was separated into pRBCs, FFP and PLTs and reconstituted into the ratios 1:1:1 and 3:1:1 at day 1, 4, 14, and 24. Standard blood count, electrolytes and coagulation proteins were quantified. The functional coagulation in ratio- and age-specific groups was evaluated using rotational thromboelastometry (ROTEM).

**Results:**

Several coagulation factors reduced significantly in the 3:1:1 ratio and were consistent with increased INR, decelerated clot formation times and A10 (amplitude 10 minutes after clotting time (CT)), flattened α-angle during the EXTEM and diminished MCF for distinct time points during the INTEM, FIBTEM and APTEM assays. With rising age of pRBCs the pH, sodium and potassium reached non-physiological levels.

**Conclusion:**

Under standardized *in vitro* conditions the higher amount of pRBCs in the 3:1:1 ratio diluted coagulation factors significantly on the expense of its functional coagulation capacity as revealed by ROTEM results. Thus, the coagulation functionality of the 1:1:1 ratio predominated.

## Introduction

As there is less doubt about the components to be administered during transfusion the evidence for an appropriate transfusion ratio is still lacking [1]. Different ratios of pRBCs, FFP and PLTs are applied in clinical practice [2-4] at which the 1:1:1 ratio has been adopted by trauma centers worldwide for the acute treatment of bleeding trauma patients [5,6]. For massive transfusion the use of a RBC:FFP ratio of 1:1 or 2:1 was recommended based on systematic reviews [7-11] and a reduction of death risk for trauma patients was associated when transfused in the range of 1:1 to 2.5:1. Currently, the PROPPR study as a phase III prospective randomized trial was conducted by several trauma centers in the United States that targets the efficacy of the transfusion ratio 1:1:1 compared to 2:1:1 (pRBCs:FFP:PLTs) within a massive transfusion (MT). Overall limited evidence due to retrospective or unfinished prospective designs [12] and numerous potential confounders [1,10,13-16] as well as poor compliance to MT protocols (MTP) during resuscitation limits the expressiveness and results of these studies.

Besides transfusion ratios the application of blood products stored less than two weeks (fresh blood) or more than 14 days (old blood) is also crucially discussed. Previously, lower postoperative complications and increased long-term survival were observed after cardiac surgery when transfusing fresh compared to older blood [17,18]. A higher mortality rate and severity of transfusion related acute lung injury (TRALI) was shown after administering stored pRBC in different animal studies [18,19] with comparable results in additional studies [20-24]. Furthermore the erythrocyte cell membrane deformability irreversibly decreased with storage duration and might be responsible for reduced oxygen transport [24-29].

The present study addressed the question of a predominance of the ratios 1:1:1 and 3:1:1 of pRBCs, FFP and PLTs in contrast to whole blood on the one hand and age-specificities of fresh and old blood on the other. For this purpose, they were compared *in vitro* under standardized conditions by measuring electrolytes, blood and coagulation proteins as well as analyzing functionality of coagulation and at distinct time points.

## Methods

### Blood donation and further processing

After institutional review board approval and written informed consent a volume of 450 ml type A blood of six voluntary donors was collected in standard bags supplemented with 63 ml of CPD (citrate, phosphate, dextrose) stabilization solution. Initially, the blood was stored for 20 hours at validated CompoCool plates (Fresenius Kabi, Bad Homburg, Germany) prior to leukocyte-depletion (by filtering) and separation (by centrifugation) into pRBCs, FFP and PLTs. The pRBC of 260 ± 50 ml volume contained 100 ml of SAG-M (adenine, glucose and mannitol) solution and were stored at 4°C ± 2°C until usage. FFP was portioned in 250 ± 30 ml bags, shock-frozen and stored at −30°C. Due to the short storage life a maximum four days old PLTs bag of 260 ± 40 ml was used that had been incubated under constant agitation at 22°C ± 2°C.

**Figure 1:**
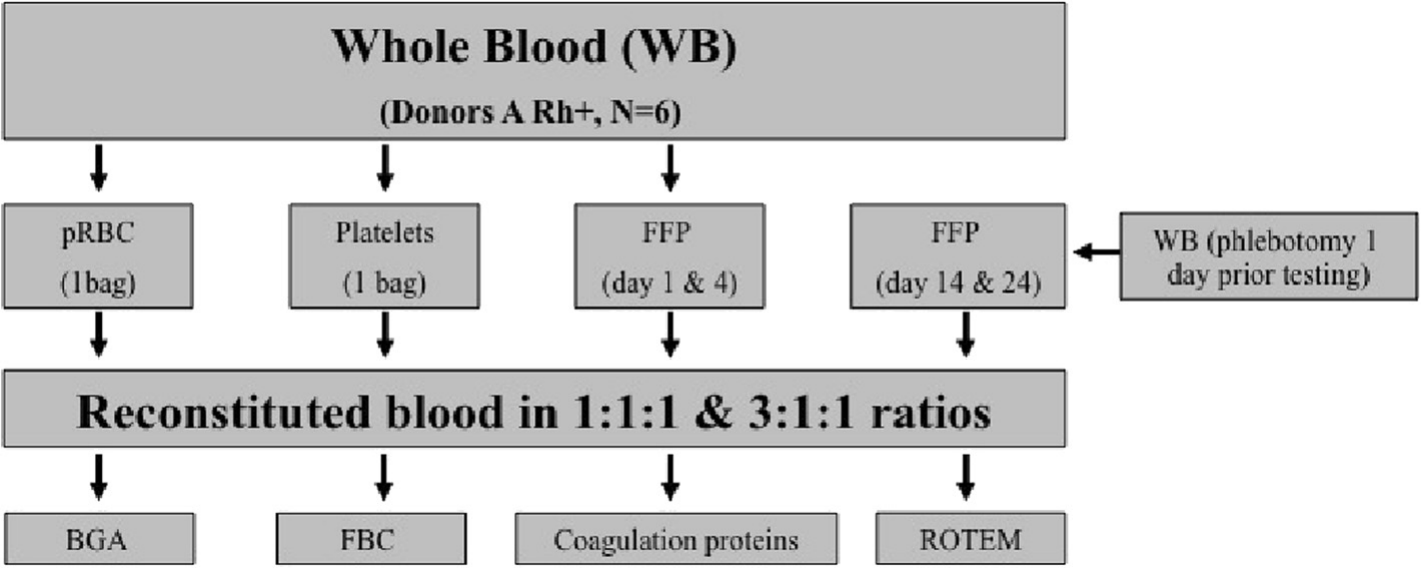
**Procedure of blood separation and reconstitution as well as subsequent experiments.**

Since the volume of the blood product bags differed, the pRBCs, FFPs and PLTs were weighted first before calculating its percentage of volume prior reconstitution which allowed a realistic transfusion procedure of the ratios 1:1:1 and 3:1:1 to a final volume of 50 ml. All measurements were performed at day one, four, 14, and 24. Additionally, whole blood was measured at day one. The detailed proceeding was represented in Figure 1.

### Measurement of blood parameters

All measurements were performed at the Institute of Transfusion Medicine, Cologne-Merheim Medical Centre. The standard hemogram (Full Blood Count, FBC) parameters erythrocytes, leucocytes, platelets, hemoglobin, hematocrit, mean corpuscular volume (MCV), mean corpuscular hemoglobin (MCH), mean corpuscular hemoglobin concentration (MCHC), and red blood cell distribution width (RDW) were measured on the Cell-Dyn 1700 Hematology Analyzer (Abbott Laboratories, Abbott Park, USA). International normalized ratio (INR), partial thromboplastin time (PTT), fibrinogen, antithrombin, the factors II, V, VII, VIII, IX, X, XI, XII, and VIII, protein C, protein S, d-dimer, vonWillebrand factor activity (vWF:a), and vonWillebrand factor Antigen (vWF:Ag) were quantified following the manufacturer’s manuals (Instrumentation Laboratory Company; Bedford, USA; Siemens Healthcare Diagnostics Products GmbH, Marburg, Germany) by using the ACL TOP system (Instrumentation Laboratory Company; Bedford, USA) and the APACT 4S Plus (Rolf Greiner BioChemica, Flacht, Germany).

The values of sodium, potassium, calcium, and the pH were determined by blood gas analysis via the IRMA TruPoint Blood Analysis System (Keller Medical GmbH, Bad Soden, Germany).

### ROTEM® analysis

**Figure 2:**
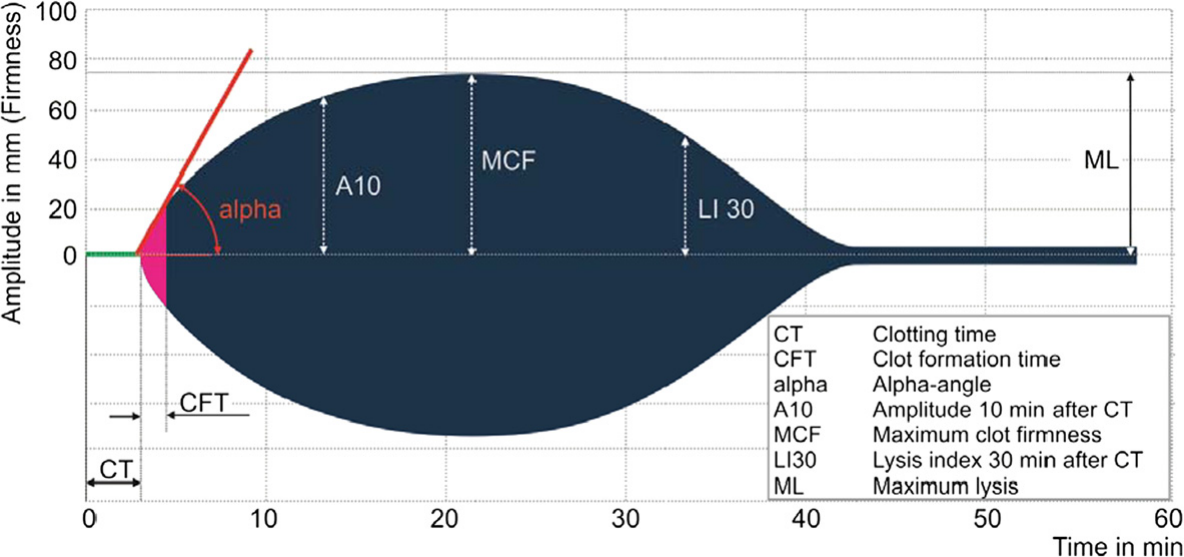
**Kinetics of hemostasis as demonstrated by a viscoelastic test (ROTEM, Tem Innovations GmbH).**

Kinetics of hemostasis was followed by ROTEM *delta* analysis in accordance to the manufacturer’s instructions (Tem International GmbH, Munich, Germany) (Figure 2). The 30 minutes thromboelastometric measurement included the extrinsically activated assay with tissue factor (EXTEM), the intrinsically activated test using ellagic acid (INTEM), the extrinsically activated test with tissue factor and the platelet inhibitor cytochalasin D (FIBTEM) as well as the extrinsically activated assay after blocking hyperfibrinolysis by aprotinin (APTEM). The parameters clotting time (CT), clot formation time (CFT), α-angle, amplitude 10 minutes after CT (A10), and maximal clot firmness (MCF) were analyzed for the EXTEM, INTEM and APTEM assays while the CFT was not determined for FIBTEM.

### Statistics

Collected data were analyzed by the nonparametric Kruskal-Wallis test to assess the effects of time and ratio in the different time-by-ratio groups using the IBM SPSS Statistics, version 21.0 (Armonk, NY, USA). *P* values were corrected in the subsequent pairwise comparison of groups. Differences were considered statistically significant at *p* < 0.05.

The cumulative comparison of coagulation factor activity was performed by calculating the overall mean of all factors for the WB, 1:1:1 and 3:1:1 group, respectively.

## Results

### Blood count and electrolytes

Irrespective of blood product age, several measured parameters differed between the ratios 1:1:1 and 3:1:1 (Table 1). The quantity of erythrocytes was significantly lower in the 1:1:1 group compared to WB. Platelets were elevated in the 1:1:1 ratio at all points in time and differed significantly from the corresponding levels of the whole blood. Leucocyte depletion of the original blood samples resulted in a drastically decline in both ratios (<500/μl). While hemoglobin and hematocrit levels of the 3:1:1 ratio and the WB were likewise, the 1:1:1 ratio had significantly reduced levels in comparison with both groups at the reviewed time points. Overall, age-specific differences were not observed for erythrocytes, platelets, hemoglobin, and hematocrit. Similarly, MCV, MCH and RDW remained constant and were not affected by age or ratio.

**Table 1:** **Significant ratio**-**specific differences of the ratios 1**:**1**:**1 and 3**:**1**:**1**

**Parameter**	**Day 1**			**Day 4**		**Day 14**		**Day 24**	
	**WB**	**1:1:1**	**3:1:1**	**1:1:1**	**3:1:1**	**1:1:1**	**3:1:1**	**1:1:1**	**3:1:1**
Erythrocytes [×10^6^/μl]	3.79 ± 0.29	2.14 ± 0.54^+^	3.65 ± 0.85	2.01 ± 0.14^*^	3.68 ± 0.29	1.96 ± 0.13^*^	3.3 ± 0.34	2.09 ± 0.64^*^	3.08 ± 0.61
Leukocytes [×10^3^/μl]	6.55 ± 2.05	0.25 ± 0.05^+^	0.33 ± 0.1^+^	0.37 ± 0.08	0.33 ± 0.14	0.35 ± 0.1	0.25 ± 0.08	0.45 ± 0.1	0.5 ± 0.18
Platelets [×10^3^/μl]	236.5 ± 43.1	348.5 ± 36.39^+,*^	226.5 ± 28.51	327.5 ± 22.42^*^	181 ± 44.09	359.17 ± 20.68^*^	231.5 ± 38.83	334.83 ± 37.5^*^	231.5 ± 58.11
Hemoglobin [g/dl]	11.78 ± 1.11	6.6 ± 1.67^+,*^	11.43 ± 2.54	6.2 ± 0.56^*^	11.42 ± 1.08	6.12 ± 0.5^*^	10.38 ± 1.25	6.47 ± 2.27^*^	9.76 ± 1.71
Hematocrit [%]	37.08 ± 3.21	20.85 ± 4.29^+,*^	35.7 ± 7.14	20.27 ± 1.55^*^	37.13 ± 3.19	19.5 ± 1.76^*^	32.97 ± 3.88	21.33 ± 7.26	30.92 ± 5.29
MCHC [g/dl]	31.75 ± 0.7	31.47 ± 1.27	31.9 ± 0.65	30.57 ± 0.6	30.72 ± 0.5	30.75 ± 0.18^*^	31.5 ± 0.64	30.25 ± 0.49	30.83 ± 0.7
pH	6.92 ± 0.06	7.04 ± 0.04^+^	6.95 ± 0.05	7.03 ± 0.05^*^	6.9 ± 0.04	6.91 ± 0.08^*^	6.76 ± 0.03	6.89 ± 0.03^*^	6.69 ± 0.06
Sodium [mM]	139.67 ± 2.33	142.62 ± 6.15	145.37 ± 5.14	140.48 ± 1.63^*^	142.3 ± 0.89	138.67 ± 0.81	138.43 ± 2.09	137.42 ± 0.99	136.72 ± 1.67
Potassium [mM]	3.33 ± 0.4	3.12 ± 0.38	2.83 ± 0.78	3.91 ± 0.38^*^	4.69 ± 0.35	6.72 ± 1.3^*^	9.19 ± 0.95	7.45 ± 0.48^*^	13.01 ± 0.15
EXTEM CFT [s]	101.83 ± 49.17	59.83 ± 10.94^*^	125.33 ± 27.5	54.83 ± 5.49^*^	127.67 ± 50.71	59.33 ± 16.46^*^	139.5 ± 46.87	74.33 ± 27.98	99.5 ± 34.81
INTEM CFT [s]	133.5 ± 67.16	70.33 ± 24.83	144.17 ± 99.03	55.5 ± 6.8^*^	119.5 ± 39.51	114.17 ± 94.2	166.5 ± 78.58	67 ± 34.48	92.5 ± 18.17
APTEM CFT [s]	119.17 ± 74.34	72.67 ± 22.81	108 ± 33.89	70.5 ± 16.88^*^	131.33 ± 57.6	66.83 ± 19.06^*^	133.5 ± 44.28	65.33 ± 30.74	84.33 ± 19.31
INR	1.12 ± 0.1	1.03 ± 0.05^*^	1.23 ± 0.08	1.13 ± 0.05^*^	1.52 ± 0.25	1.12 ± 0.04^*^	1.53 ± 0.15	1.17 ± 0.08^*^	1.45 ± 0.14
PTT [s]	38.33 ± 4.59	33.67 ± 3.83	36.83 ± 5.49	33.83 ± 2.79^*^	44.83 ± 6.88	38.5 ± 4.14^*^	47.67 ± 7.15	36.83 ± 4.4^*^	43.5 ± 5.32
Fibrinogen [mg/dl]	254.17 ± 51.33	226.83 ± 23.79	173.8 ± 31.3^+^	224.33 ± 21.94^*^	147.33 ± 30.74	215.17 ± 25.56^*^	143.67 ± 16.46	240.17 ± 20.47^*^	170 ± 17.12
Antithrombin [%]	77.17 ± 7.41	68.83 ± 7.78	49.17 ± 8.54^+^	64.67 ± 3.98^*^	38.17 ± 7.25	67.17 ± 3.31^*^	38.33 ± 6.12	63.17 ± 3.43^*^	41.33 ± 3.27
Factor II [%]	82.33 ± 8.33	76.33 ± 4.23	55.17 ± 7.19^+^	74 ± 5.48^*^	46.33 ± 8.71	68.5 ± 8.07^*^	43.83 ± 9.7	63.17 ± 5.34	45.67 ± 5.54
Factor V [%]	81.17 ± 7.41	72.67 ± 6.89	53 ± 10.1^+^	57.33 ± 4.41^*^	34.5 ± 6.25	58.67 ± 3.72^*^	35.83 ± 2.64	56.33 ± 6.98^*^	40.33 ± 5.09
Factor VII [%]	73.67 ± 11.33	79.83 ± 10.46^*^	56.33 ± 7.76	67.17 ± 9.15^*^	42 ± 9.27	64.67 ± 9.67^*^	41.33 ± 8.31	61.17 ± 11.41^*^	45 ± 9.92
Factor VIII [%]	95.33 ± 29.12	83.75 ± 13.35	57.83 ± 10.8^+^	71.8 ± 9.88^*^	46.17 ± 16.55	53.83 ± 11.2^*^	39.17 ± 8.8	71.67 ± 30.94	53.33 ± 23.89
Factor IX [%]	89 ± 8.2	95 ± 5.22^*^	70.67 ± 8.62	87 ± 5.33^*^	54.5 ± 10.56	74.5 ± 10.91^*^	51.17 ± 11.36	77.33 ± 16.37^*^	50.67 ± 9.14
Factor X [%]	73.5 ± 9.09	79.33 ± 5.92^*^	54.17 ± 5.88	72.33 ± 8.24^*^	48.83 ± 6.68	68.67 ± 13.98^*^	44.17 ± 11.07	71.17 ± 10.82^*^	49.83 ± 10.38
Factor XI [%]	83.8 ± 29.8	96 ± 15.3^*^	71.67 ± 6.35	91.83 ± 18.8	72.83 ± 33.88	72.67 ± 15.07^*^	49.5 ± 12.32	66.5 ± 11.78^*^	48.17 ± 8.33
Factor XII activity [%]	86.17 ± 28.27	75.67 ± 9.14^*^	54.67 ± 8.26^+^	88 ± 10.1^*^	48.33 ± 13.85	76.5 ± 22.73^*^	50.17 ± 20.44	69.67 ± 16.01^*^	48.33 ± 9.42
Factor XIII [%]	115.67 ± 11.18	100.67 ± 12.4	77.17 ± 10.67^+^	97.5 ± 11.71^*^	67.17 ± 16.04	86.33 ± 6.38^*^	65.5 ± 4.09	92.17 ± 19.05^*^	61 ± 1.79
Protein C activity [%]	71.67 ± 13.98	70.33 ± 5.96^*^	50.5 ± 4.97^+^	70.67 ± 7.42^*^	41.33 ± 8.36	60.67 ± 6.74^*^	37.33 ± 7.26	63.67 ± 5.16^*^	48.17 ± 6.97
Protein S activity [%]	89.17 ± 16.93	60.33 ± 7.12	43.17 ± 11.41^+^	33 ± 4.34^*^	21.17 ± 8.82	55 ± 8.12^*^	33.33 ± 7.39	43.8 ± 15.42	28.83 ± 12.24
v. Willebr. activation [%]	128.83 ± 39.78	97 ± 19.01	72.2 ± 15.9^+^	98.67 ± 18.35^*^	67.17 ± 23.04	67.5 ± 26.61	46 ± 14.56	82.33 ± 28.11	60.17 ± 22.11
v. Willebr. antigen [%]	141.83 ± 45.28	112.67 ± 27.81	77.2 ± 17.08^+^	114.83 ± 27.69^*^	71.67 ± 22.17	78 ± 25.87	53.17 ± 17.68	107.83 ± 39.91	76.5 ± 28.74

Values are represented as mean ± standard deviation. Differences (*p* < 0.05) are marked as + when comparing with whole blood (WB) and as *to compare both ratios irrespective of time point.

Only few age effects could be observed (Table 2) and thereof most notably the values of potassium and sodium. Independent of ratio potassium increased progressively over time being 2.4- and 4.6-fold higher after 24 days in the 1:1:1 and 3:1:1 ratio, respectively. In contrast, sodium reduced with increasing age and differed significantly in both ratios between day one and 24. The environment of the reconstituted blood slightly acidified as in both ratios the pH value decreased with age and was reduced stronger in the 3:1:1 group from day four (Table 2). No alteration of calcium was determined during the measurement.

**Table 2:** **Significant age**-**specific differences at day one**, **four**, **14**, **and 24**

**Parameter**	**1:1:1**				**3:1:1**			
	**Day 1**	**Day 4**	**Day 14**	**Day 24**	**Day 1**	**Day 4**	**Day 14**	**Day 24**
Leukocytes [×10^3^/μl]	0.25 ± 0.05^c^	0.37 ± 0.08	0.35 ± 0.1	0.45 ± 0.1	0.33 ± 0.1	0.33 ± 0.14	0.25 ± 0.08	0.5 ± 0.18
pH	7.04 ± 0.04^b,c^	7.03 ± 0.05^e^	6.91 ± 0.08	6.89 ± 0.03	6.95 ± 0.05^b,c^	6.9 ± 0.04^e^	6.76 ± 0.03	6.69 ± 0.06
Sodium [mM]	142.62 ± 6.15^c^	140.48 ± 1.63^e^	138.67 ± 0.81	137.42 ± 0.99	145.37 ± 5.14^b,c^	142.3 ± 0.89^e^	138.43 ± 2.09	136.72 ± 1.67
Potassium [mM]	3.12 ± 0.38^b,c^	3.91 ± 0.38^e^	6.72 ± 1.3	7.45 ± 0.48	2.83 ± 0.78^b,c^	4.69 ± 0.35^e^	9.19 ± 0.95	13.01 ± 0.15
INTEM CT [s]	204.83 ± 13.6^c^	229.33 ± 28.51	243.5 ± 121.51	284.33 ± 67.57	206.83 ± 16.46	200.83 ± 14.93^e^	245 ± 84.52	259.83 ± 25.86
FIBTEM CT [s]	57.83 ± 10.65	108.33 ± 91.24	69.5 ± 38.9	46.17 ± 29.17	84.33 ± 15.72^c^	52.33 ± 26.24	132.33 ± 107.69	45.17 ± 19.09
INR	1.03 ± 0.05^c^	1.13 ± 0.05	1.12 ± 0.04	1.17 ± 0.08	1.23 ± 0.08 b	1.52 ± 0.25	1.53 ± 0.15	1.45 ± 0.14
Factor II [%]	76.33 ± 4.23^c^	74 ± 5.48	68.5 ± 8.07	63.17 ± 5.34	55.17 ± 7.19	46.33 ± 8.71	43.83 ± 9.7	45.67 ± 5.54
Factor V [%]	72.67 ± 6.89^a,c^	57.33 ± 4.41	58.67 ± 3.72	56.33 ± 6.98	53 ± 10.1^a,b^	34.5 ± 6.25	35.83 ± 2.64	40.33 ± 5.09
Factor VII [%]	79.83 ± 10.46^c^	67.17 ± 9.15	64.67 ± 9.67	61.17 ± 11.41	56.33 ± 7.76	42 ± 9.27	41.33 ± 8.31	45 ± 9.92
Factor IX [%]	95 ± 5.22^b^	87 ± 5.33	74.5 ± 10.91	77.3 ± 16.37	70.67 ± 8.62^c^	54.5 ± 10.56	51.17 ± 11.36	50.67 ± 9.14
Factor XI [%]	96 ± 15.3	91.83 ± 18.8	72.67 ± 15.07	66.5 ± 11.78	71.67 ± 6.35^b,c^	72.83 ± 33.88	49.5 ± 12.32	48.17 ± 8.33
Factor XIII [%]	100.67 ± 12.4	97.5 ± 11.71	86.33 ± 6.38	92.17 ± 19.05	77.17 ± 10.67^c^	67.17 ± 16.04	65.5 ± 4.09	61 ± 1.79
Protein C activity [%]	70.33 ± 5.96	70.67 ± 7.42	60.67 ± 6.74	63.67 ± 5.16	50.5 ± 4.97^b^	41.33 ± 8.36	37.33 ± 7.26	48.17 ± 6.97
Protein S activity [%]	60.33 ± 7.12^b^	33 ± 4.34	55 ± 8.12	43.83 ± 15.42	43.17 ± 11.41^a^	21.17 ± 8.82	33.33 ± 7.39	28.83 ± 12.24
D-dimer [μg/l]	216.6 ± 48.43^b^	239.5 ± 43.98^d^	123 ± 26.78	156 ± 50.45	185.2 ± 36.64	180.67 ± 37.6	119.5 ± 17.04	150.5 ± 32.44

Values are represented as mean ± standard deviation. Lowercases indicate significant differences (*p* < 0.05) between: a - day 1 and 4, b - day 1 and 14, c - day 1 and 24, d - day 4 and 14, e - day 4 and 24, and f - day 14 and 24, respectively.

### Coagulation proteins

**Figure 3:**
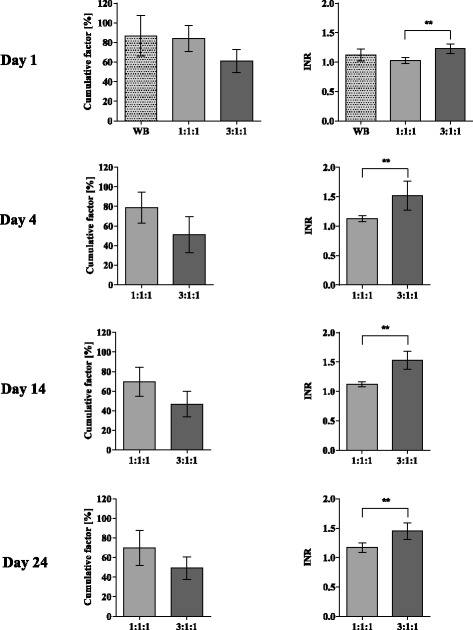
**Quantitative cumulative factor quantity (summing up coagulation factors FII, FV, FVII, FVIII, F IX, FX, FXI, FXII, FXIII) and INR of the WB, 1:1:1 and 3:1:1 groups.** Levels are represented as mean and standard deviation. Bars are depicted as dotted (WB), light grey (1:1:1) and dark grey (3:1:1). Significances were observed at *p < 0.05; **p < 0.01; ***p < 0.001.

For almost all coagulation factors ratio-specific reductions in the 3:1:1 group were observed at any time point when comparing both ratios (Table 1). The factors VII, IX, X and XII were constantly and significantly reduced in 3:1:1 samples, independent of age while the factors II, V, and XIII significantly declined not before day four. Similar to the blood count parameter the quantity of coagulation factors was affected minor by age (Table 2). Although several factors tended to be reduced after 24 days only the factors II, V and VII measured in the 1:1:1 ratio and factors IX, XI and XIII in the 3:1:1 group reached the level of significance. The cumulative factors were comparable in the WB and 1:1:1 group whereas a precise decline was observed when comparing both groups to the 3:1:1 ratio at day 1 or when comparing the 1:1:1 with the 3:1:1 ratio at the respective time points (Figure 3).

The activity of the coagulation inhibitors antithrombin and protein C as well as its cofactor protein S were significantly lowered in the 3:1:1 compared to WB (day one) and in most cases considerably reduced in 1:1:1 ratio at all ages (Table 2). Fibrinogen was also diluted in 3:1:1 samples from day four on (Table 1).

### Functionality of coagulation

**Figure 4:**
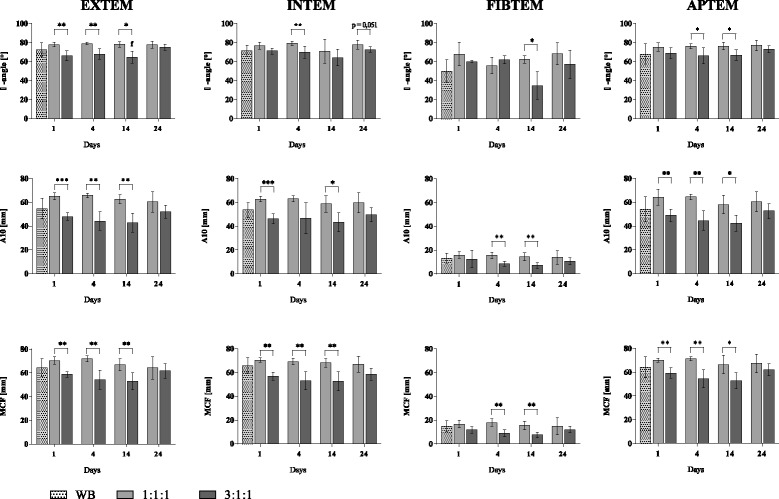
**Functional characterization of coagulation via ROTEM analysis.** The α-angle, amplitude after 10 minutes (A10) and maximum clot firmness (MCF) after 30 minutes of the EXTEM, INTEM, FIBTEM, and APTEM assays are represented as mean and standard deviation. Bars are depicted as dotted (WB), light grey (1:1:1) and dark grey (3:1:1). Significances were observed at *p < 0.05; **p < 0.01; ***p < 0.001. Age differences (*p* < 0.05) were indicated with the lowercases a (day 1 ↔ 4), b (day 1 ↔ 14), c (day 1 ↔ 24), d (day 4 ↔ 14), e (day 4 ↔ 24), and f (day 14 ↔ 24), respectively.

Consistent with the reduction of coagulation proteins in the 3:1:1 ratio an elevated PTT and INR were observed in the 1:1:1 group irrespective of age. Both values reached significant levels with the exception of PTT at day one (Table 1). Furthermore, the INR trended to increase with age in both ratios. A declined functionality of the extrinsic and intrinsic system was also measured by ROTEM analysis. The CFT significantly decelerated at almost all time points in the 3:1:1 group of the EXTEM approach (Table 1). Similarly, CTs were prolonged by trend in the 3:1:1 group, however, not reaching significant level. Also the α-angle and the clot amplitudes after 10 and 30 minutes declined in the 3:1:1 ratio at day one, four and 14 (Figure 4).

For the INTEM, FIBTEM and APTEM assays the main difference were observed after four and 14 days (Table 1, Figure 4). Thereafter mostly tendencies were determined due to a high variation within the groups. However, the MCF seemed to be the most consistent value in these three approaches providing significant declines in the 3:1:1 ratio irrespective of the activation pathway. As observed for other measured values the age differences were inconsistent and being present for few comparisons of EXTEM α-angle as well as for INTEM and FIBTEM CTs (Table 2).

## Discussion

### The ratio concept

Over 10 years ago the correlation of transfusion practices and patients’ outcome was stronger focused in trauma research. Several publications [2,10,11,30] underlined the superiority for using defined ratios of pRBCs and FFPs solely or combined with PLTs. Due to heterogeneity such as patients’ inclusion criteria (if they were even defined) and in the applied ratios (1:1; 2:1, 3:1 etc.) the evidence of these studies was limited. In 2007, Borgman et al. [2] firstly presented a systematic analysis of trauma care including a transfusion ratio concept that increased the survival rate in major bleeding patients. This study still represents the unproven guideline in handling these kinds of seriously injured patients. Currently, the multicenter PROPPR study systematically compared the transfusion ratios 1:1:1 and 2:1:1 (pRBCs:FFP:PLTs) in a clinical trial and brought first (but final) evidence in the discussion regarding transfusion ratios [31].

In this *in vitro* study the physiological characteristics of the 3:1:1 ratio go along with a reduction of all coagulation factors resulting in a lowered hemostasiological potential that was indicated by elevated INR and viscoelastic tests. The thrombelastometric findings (ROTEM) in dilution effects in the 3:1:1 ratio became evident by a decreased dynamic clot formation and clot quality as indicated by a flattened α-angle and a lowered clot firmness after 10 and 30 minutes (A10, MCF). Furthermore, a trend of decelerated clot formation could be observed.

There was no evidence that the processing of plasma (freezing, storage, thawing) may explain the changes in factor quantity and viscoelastic functionality of the 3:1:1 group, since 10 days of storage and subsequent thawing did not affect coagulation factors as shown previously [32]. In addition, this proceeding effect would have been observed in the 1:1:1 ratio as well. To ensure the allocation of functional factors in FFPs and to further minimize influencing effects on coagulation, maximum 4 days old factor-rich plasma was used at any time point before reconstitution. Thus, the lower quantity of coagulation factors whether significantly reduced in the 3:1:1 ratio or trend to be decreased can rather be explained by diluting effects than by inactivation of factors. Furthermore, cumulative factors were comparable between WB and 1:1:1 providing an adequate initial basis for coagulation [30,33]. In summary the hemostasiological potential of the 1:1:1 ratio rather referred to that of WB. Transfusion ratios leading to a nearly physiological hemostasiological potential and an effective coagulation may reduce the amount of pRBCs being transfused. According to the current European guideline [34] transfusion of pRBC is recommended in patients with Hb values below 7 mg/dl.

The findings of the present study were in line with results of a recently published *in vitro* study in which a dilution effect in a 3:1 (pRBC:FFP) ratio was determined by simulating postpartum hemodilutional coagulopathy [35]. Therefore, there seemed to be a superiority of the 1:1 ratio of pRBC and FFP transfusion optimized by an additional application of the same ratio of PLTs. Interestingly, no dilution effect due to FFP was observed though not only concentrates of clotting factors were used. Similar results were reported for a thrombelastography (TEG) approach in which different ratios of pRBC:FFP:PTL were analyzed in vitro. In line with the results of the present study, a higher proportion of pRBCs such as 8:4:1 of pRBC:FFP:PTLs resulted in decreased abilities to form a stable clot (reduced angle and maximal amplitude) [36].

This *in vitro* experiment enabled the assessment of quantitative and functional coagulation under standardized environments and revealed main findings in dilution and associated effects on functional coagulation. However, interrelated physiological impact on TIC like hypothermia, acidosis, inflammation, shock or the preliminary treatments such as pre-hospital volume administration [37-39] and therefore, dilution and hyperpermeability, shedding of the endothelial glycocalyx with autoheparinization [40] and activation of the protein C pathway [41,42] influence its coagulation potency. Disease pattern leading to major bleeding are very individual. Therefore, we could only propose a possible influence of the applied ratio of blood products in human. Efficient therapy of coagulopathy reduces significantly morbidity and mortality in trauma patients [2,43]. Independent from (surgical) therapies, coagulopathy is recognized as independent disease with an individual need for therapy nowadays [44,45].

### Age of blood products

Addressing the question if there are differences in newer compared to older blood trends of reduced corpuscular components after 24 days were shown. Inverse the highest potassium and lowest pH value were measured at day 24 which might be explained by an increasing erythrocytes degradation [46]. With regard to clinical practice one should consider that potassium levels higher than 8 mmol/l have to be taken into further account when applying the 3:1:1 ratio. As demonstrated in previous clinical studies [27-29,47,48] in which the length of storage of pRBC influenced hemolysis, changes in deformability as well as osmotic fragility the results of this study did not reveal any continuous age effect according to the hemostasiological potential. However, the changes of potassium (13.01 ± 0.15 mM), sodium (136.72 ± 1.67 mM) and pH levels (6.69 ± 0.06) associated with aging blood products cannot be disregarded when applying to patients after 24 days of storage.

Regarding clotting time and clot formation a continuous but not significant increase was detected for INTEM while FIBTEM values reacted vice versa.

## Conclusions

This systematic *in vitro* study offers valuable information about transfusion ratios as well as influencing age effects on coagulation. With regard to limitations of *in vitro* trials, the presented results suggest a superiority of 1:1:1 ratio in a physiological setting in case of transfusion encouraging higher use of plasma rather than pRBCs. Thus, dosage, ratio and administration of blood components still remain a dichotomy between benefits and harms whereby this study highlighted the need for multi-center clinical trials to determine the optimal transfusion strategy.

### Key messages

- The higher proportion of pRBCs of the 3:1:1 ratio resulted in dilution properties of almost all coagulation factors and inhibitors.
- Dilution likewise affected the hemostasiological potential which was indicated by an elevated PTT and INR in the 3:1:1 compared to 1:1:1 group.
- The reduced functional coagulation of the 3:1:1 ratio was further specified by a decelerated CFT, flattened α-angle and diminished MCF of the ROTEM analysis.

## Abbreviations

ACT: Activated clotting time
APTT: Activated partial thromboplastin time
BGA: Blood gas analysis
CFT: Clot formation time
Functional fibrinogen: Tissue factor–activated thromboelastography including a platelet inhibitor
FBC: Full blood count
FFP: Fresh frozen plasma
INR: International normalized ratio
MA: Maximum amplitude
MCF: Maximum clot firmness
MTP(s): Massive transfusion protocol(s)
PLT(s): Platelet concentrate(s)
pRBCs: packed red blood cells
TEG: Thromboelastography
WB: Whole blood
